# Assessing Performance and Engagement on a Computer-Based Education Platform for Pharmacy Practice

**DOI:** 10.3390/pharmacy8010026

**Published:** 2020-02-24

**Authors:** Kelly Grindrod, Katherine Morris, Rosemary Killeen

**Affiliations:** 1 School of Pharmacy, University of Waterloo, Waterloo, ON N2L 3G1 Canada; rosemary.killeen@uwaterloo.ca; 2 Information and Data Management, Ontario College of Pharmacists, Toronto, ON M5R 2R4, Canada; kmorris@ocpinfo.com

**Keywords:** continuing professional development, computer-based education, pharmacy practice, online education

## Abstract

A computer-based education platform was developed using a theory-based approach to help Canadian pharmacy professionals adopt their full scope of practice. Data from the platform were used to identify factors that impacted user performance and engagement. A de-identified dataset included response data for 21 unique modules, including quiz responses and self-reflection questions. Outcome measures included user performance (mean quiz score) and engagement (completion rate for attempted modules). Analysis of variance (ANOVA), multivariate regression modelling, and machine learning cluster analysis were used to analyze the data. Of the 5290 users, 68% were pharmacists, 11% were technicians, 13% were pharmacy students, and 8% were pharmacy technician students. Four clusters were identified separately for pharmacists and technicians. Clusters with the higher performance and engagement tended to have more users practicing in community pharmacies while the lower performing clusters tended have more internationally trained users. In the regression modelling, pharmacists performed better than technicians and students while students were more engaged (*p* < 0.0001). Further, internationally trained pharmacists had slightly lower scores but similar engagement compared to domestically trained pharmacists (*p* < 0.0001). Users demonstrated higher performance on modules related to scope of practice than on clinical topics, and were most engaged with topics directly impacting daily practice such as influenza vaccinations and new and emerging subjects such as cannabis. The cluster analysis suggests that performance and engagement with a computer-based educational platform in pharmacy may be more related to place of practice than to personal demographic factors such as age or gender.

## 1. Introduction

The scope of pharmacy practice continues to expand worldwide, especially in areas such as the administration of injections, smoking cessation consulting, medication reviews, and pharmacist prescribing [1]. Research has shown that pharmacists often consider an expanding scope of practice to be a “legitimization” of prior practices and are more likely to offer a new service if they were already doing it in a less formal manner, although there can be a tension between a more professional role and the day-to-day need for technical efficiency [2,3,4,5]. The challenge, however, is that new services associated with an expanding scope can have low uptake, may be delivered in ways that do not align with policy, or may be preferentially offered to less complicated patients [6,7,8,9]. While current methods of continuing professional development commonly used in Canada, such as conference attendance and home study units, are still deemed valuable, they may be insufficient to facilitate widespread practice change [10]. Very little research has been done to date on the use of computer-based education for helping pharmacists close these gaps. Generally, mobile- and computer-based digital education can improve knowledge and skills in ways that are similar to traditional education [11,12,13]. Advantages of computer-based learning are that it can be more interactive [14], that it allows users to ability to apply new knowledge to diverse cases [15], and that it can be accessible to a wider range of healthcare providers such as those in rural practice [16]. That said, there are also significant concerns that computer-based education requires more self-discipline or motivation, which can lead to lower engagement, overall [17].

Pharmacy5in5 was launched in January 2018. It is a computer-based learning platform that aims to help Canadian pharmacy professionals build their knowledge and skills related to the expanding scopes of practice. The development of the platform has been described elsewhere [18]. Briefly, it is an online education platform that can be used on a computer or mobile device. It has a theory-based design that uses case-based quizzes and multimedia resources to apply knowledge to real-life scenarios, while also allowing users to reflect on their past behavior and to compare themselves to their peers. In developing the platform, one of the challenges was to ensure that a variety of users could interact with the content and a diverse audience would find it engaging. For example, pharmacists, students, and technicians may all experience the platform differently, as could those working in community pharmacy or primary care, those who have more or less practice experience, or those who trained domestically or internationally. Thus, the objective of this paper was to identify factors that impact user performance and engagement on a computer-based education platform for pharmacy professionals in Canada.

## 2. Materials and Methods 

All users of the platform provide consent to the secondary use of their de-identified data for research purposes. The study was conducted in accordance with the Declaration of Helsinki, and the protocol was approved by a University of Waterloo ethics committee (ORE#22642). This paper reports on data from the platform launch in January 2018 to November 2019.

Population: The computer-based education platform is freely available to all pharmacy professionals in Canada. On registering, users provide information about their general demographics and work environment and consent to the secondary use of de-identified data for research and development. For this analysis, data were included for all users who self-identified as pharmacists, pharmacy technicians, pharmacy students, or pharmacy technician students in Canada. Pharmacists and technicians who indicated that they were not registered with a pharmacy regulatory body in Canada (e.g., unlicensed pharmacist) were excluded. Users involved in the development of the module were also excluded.

Dataset: A de-identified dataset was downloaded from the platform and cleaned to remove duplicate data and test accounts. For each user, the dataset included age, gender, years in practice, user type (pharmacist, technician, pharmacy student, technician student), geographic region, main practice location (primary care, hospital, independent community, large chain community, small chain/banner community, and university/academic), and place of training (domestic, international, both). The dataset also included user response data for all quizzes attempted and the date a module was started and completed. Finally, the dataset included reflection question responses, where users answered questions about their past behaviors (e.g., “Over the past 3 months, have you contacted a prescriber with a concern about the safety of an opioid prescription?” or “Have you ever adapted the dose of a medication (increased or decreased the dose?”).

Outcomes: The first outcome measure was performance, which was measured by the overall quiz score mean, with each quiz having a raw score out of five. The second outcome measure was engagement, which was measured by the overall number of quizzes completed. The third outcome measure was persistence, which was measured by the overall proportion of quizzes completed in a module attempted (out of a possible seven quizzes per module) and the proportion of modules attempted (out of a possible 21 modules). Persistence was calculated as:

Persistence = (overall proportion of quizzes completed + proportion of modules attempted) * 50,

where the multiplication factor of 50 is meant to provide an easy to understand scale from 0 to 100.

Analysis: The R software package was used for all data analysis (R Foundation for Statistical Computing, Version 3.6.1). In this paper, the focus was on the data related to the first attempt of any quiz. The analysis was divided into two main areas: (1) Finding natural clusters of performance and engagement; and (2) understanding the relationship between performance, engagement, and user demographics. 

Cluster Analysis: To identify clusters, a “cluster analysis” was performed, which is a machine learning technique that creates “unsupervised clusters”. The goal of the cluster analysis was to understand the underlying structure in the engagement and performance metrics. Pharmacists and pharmacy technicians were analyzed separately. Four metrics were used to cluster the data: Overall quizzes completed; overall mean quiz score; overall proportion of quizzes completed; and number of modules attempted (modules where at least one quiz was completed). The Hopkins statistic [19] was initially used to identify if the data were clusterable, and the partitioning around medoids (PAM) was used to create clusters [20,21]. The Calinski–Harabasz criterion was used to identify the optimal number of clusters [22]. Bootstrapping by re-sampling was used to evaluate the stability of the clusters, which relied on the Jaccard co-efficient (a similarity measure between sets) to determine how well the clusters behaved [23,24]. Jaccard coefficient values greater than 0.5 indicate a stable solution.

Relationships between demographics and performance and engagement: Multivariate regression modelling was used to determine the relationship between the outcome measures (performance, engagement, and persistence), demographics, and self-reported past behavior. To identify specific differences within variables, analysis of variance (ANOVA) models were used followed by Tukey’s honest significance differences test to identify specific differences. It was hypothesized that both performance and engagement would be higher for pharmacists compared to other user types (students and technicians), for domestically trained pharmacists (compared to internationally trained pharmacists), and for users with >10 years in practice (compared to users with <10 years in practice). It was also hypothesized that users with poorer performance would have less engagement with the platform. Finally, it was hypothesized that users who self-reported they had performed a behavior in the past three months would be more likely to complete the associated quiz and would have a higher performance. 

## 3. Results

The dataset contained data for 5290 users, which included 3579 (68%) pharmacists, 595 (11%) technicians, 711 (13%) pharmacy students, and 405 (8%) pharmacy technician students (Table 1). Of 3739 users (71% of total) were female, the average age was 41 years, and users had practiced for a median of eight years. Of the pharmacists included, 2434 (68%) received their entry-to-practice training in Canada. Of the 861 (24%) pharmacists who received their initial training outside Canada, 284 (32%) had also received training in Canada. The dataset included users from across Canada, but most were from Ontario (4288, 81%), followed by Alberta (451, 9%), and British Columbia (125, 2%).

### 3.1. Cluster Analysis

#### 3.1.1. Pharmacist Cluster Analysis

**Figure 1:**
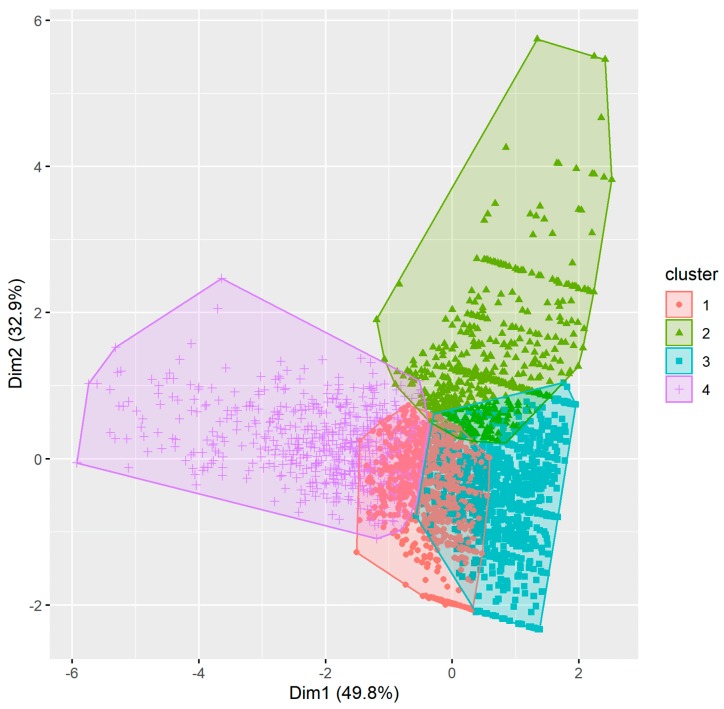
Clusters using the partitioning around medoids (PAM) approach for pharmacists.

**Figure A1:**
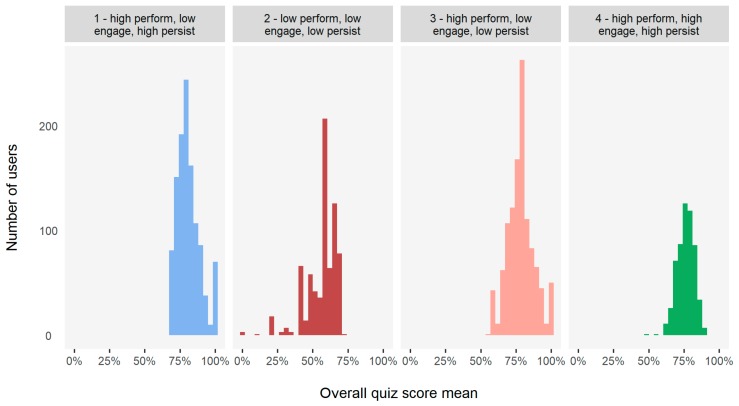
Histogram of the distribution of the mean overall pharmacist quiz score (performance) in the four clusters.

**Figure A2:**
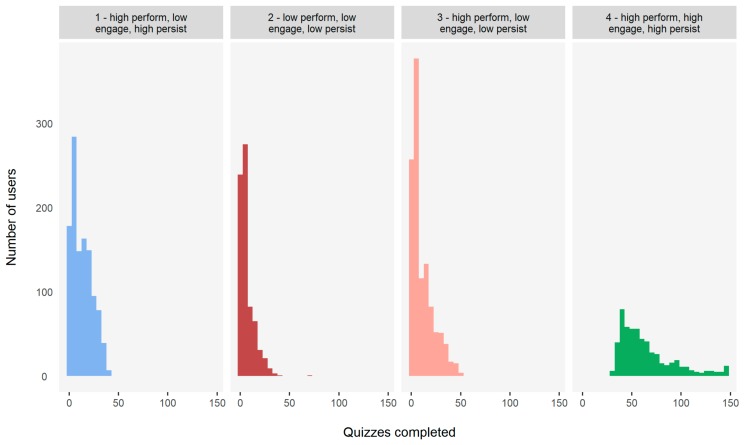
Histogram of the distribution of the number of quizzes completed (persistence) by pharmacists in the four clusters.

**Figure A3:**
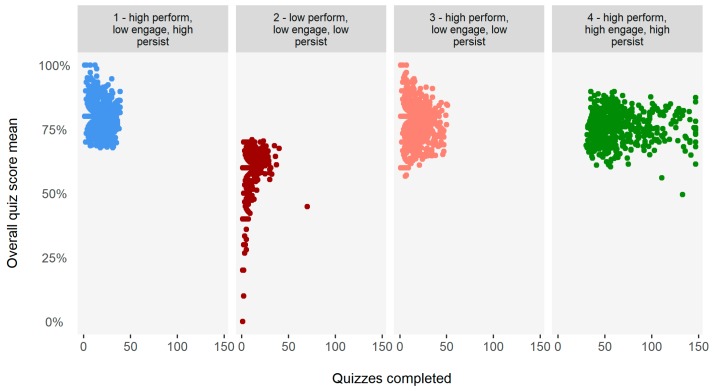
Scatterplot of the distribution of the mean overall quiz score (performance) and the number of quizzes completed (persistence) in the four pharmacist clusters.

The Hopkins statistic (0.06) indicated that the pharmacist metrics data were clusterable. Using the Calinski–Harabasz criterion, four clusters were identified (Figure 1). The Jaccard coefficient values corresponding to each cluster (0.98, 0.93, 0.87, 0.93) were greater than 0.5, indicating that the four-cluster solution was stable. The pharmacist clusters were defined as follows using the three outcome measures metrics of performance, engagement, and persistence (more information in Appendix A, Figure A1, Figure A2 and Figure A3):

- Cluster 1: High performance, low engagement, high persistence;
- Cluster 2: Low performance, low engagement, low persistence;
- Cluster 3: High performance, low engagement, low persistence;
- Cluster 4: High performance, high engagement, high persistence.

In terms of demographics, the clusters had similar age, gender, and education profiles (Table 2). Cluster two had the highest proportion of internationally trained pharmacists while cluster four had the highest proportion of community pharmacists.

#### 3.1.2. Technician Cluster Analysis

**Figure 2:**
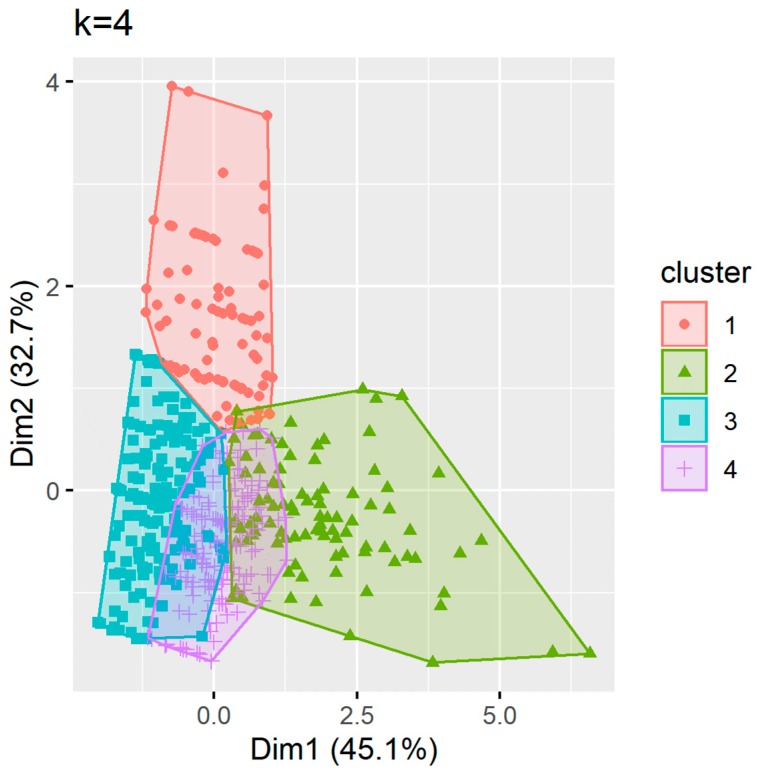
Clusters using the partitioning around medoids (PAM) approach for technicians.

**Figure A4:**
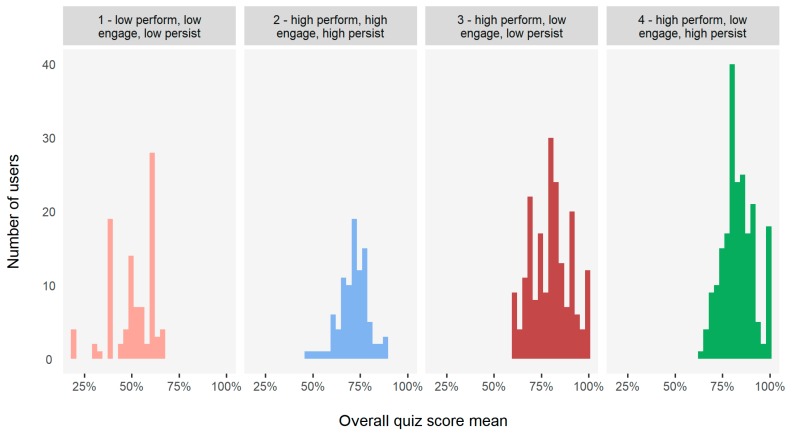
Histogram of the distribution of the mean overall technician quiz score (performance) in the four clusters.

**Figure A5:**
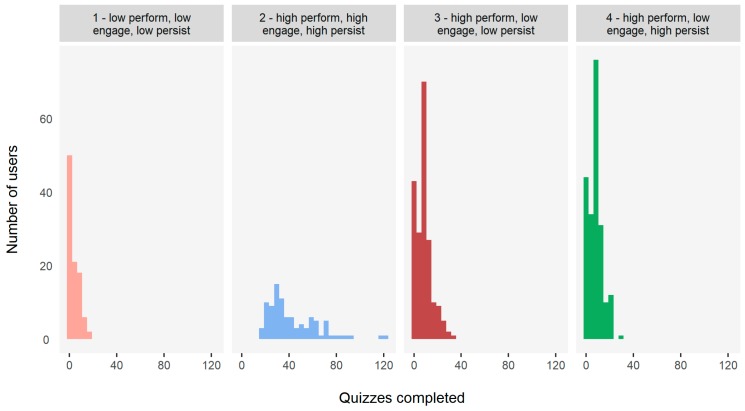
Histogram of the distribution of the number of quizzes completed (persistence) by technicians in the four clusters.

**Figure A6:**
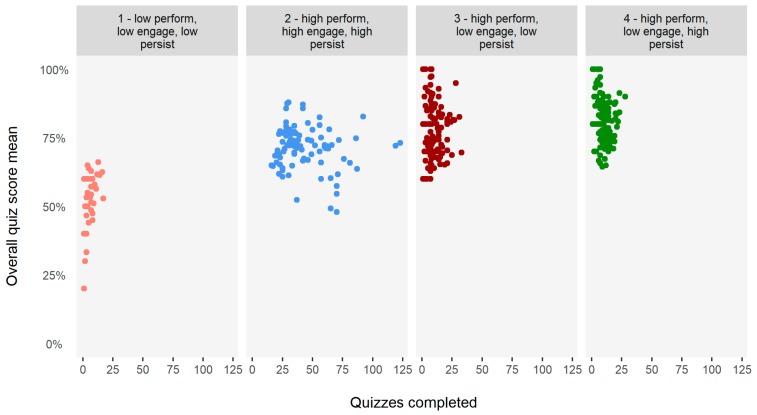
Scatterplot of the distribution of the mean overall quiz score (performance) and the number of quizzes completed (persistence) in the four technician clusters.

For technician data, the Hopkins statistic (0.12) indicated that the technician metrics data were clusterable. Four clusters were identified using the Calinski–Harabasz criterion (Figure 2). The Jaccard coefficient values were also greater than 0.5 (0.86, 0.72, 0.91, 0.87), indicating that the four cluster solution was stable (Figure 2). The technician clusters were defined as follows (more information in Appendix A, Figure A4, Figure A5 and Figure A6):

- Cluster 1: Low performance, low engagement, low persistence;
- Cluster 2: High performance, high engagement, high persistence;
- Cluster 3: High performance, low engagement, low persistence;
- Cluster 4: High performance, low engagement, high persistence.

Technician clusters had similar age, gender, and education profiles (Table 3). Cluster one had the lowest proportion of Canadian trained technicians while cluster two had the highest proportion of community-based technicians.

### 3.2. Regression Analysis

#### 3.2.1. Relationships between Demographics, Performance, and Engagement

**Figure A7:**
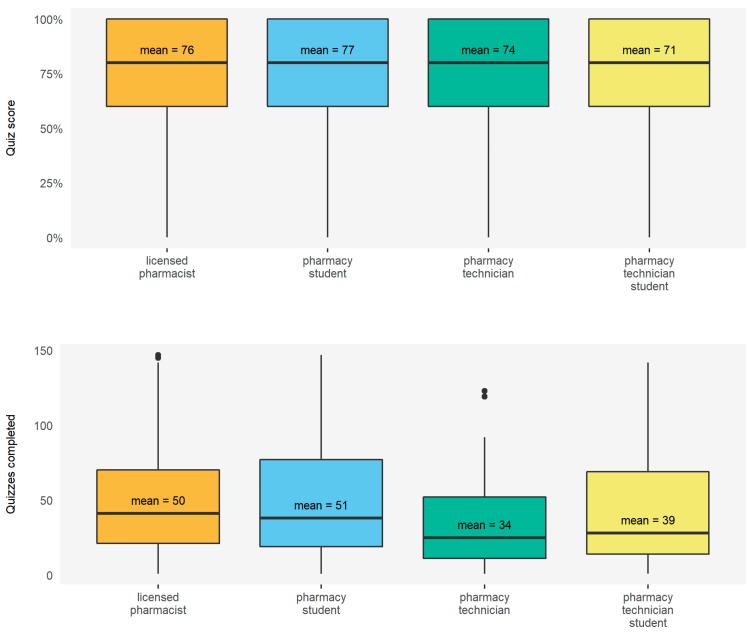
Performance and engagement by user type.

Based on the regression models, pharmacists performed better than all other user categories, while pharmacy students had the highest level of engagement (Table 4). According to the ANOVA, there were statistically significant differences between user categories for performance (F (3, 98,227) = 117.8, *p* < 0.001), and engagement (F (3, 98,227) = 623.4, *p* < 0.001). The differences in performance were small, while the differences in engagement were more pronounced (Appendix A, Figure A7). 

Users practicing in primary care performed better than other users, while engagement was highest for users working in independent practice. According to the ANOVA, there were statistically significant differences between practice locations for performance (F (6, 98,224) = 17.58, *p* < 0.001) and engagement (F (6, 98,224) = 372.2, *p* < 0.001). As above, the differences in performance were small, while the differences in engagement were larger (Appendix A, Figure A7).

**Figure A8:**
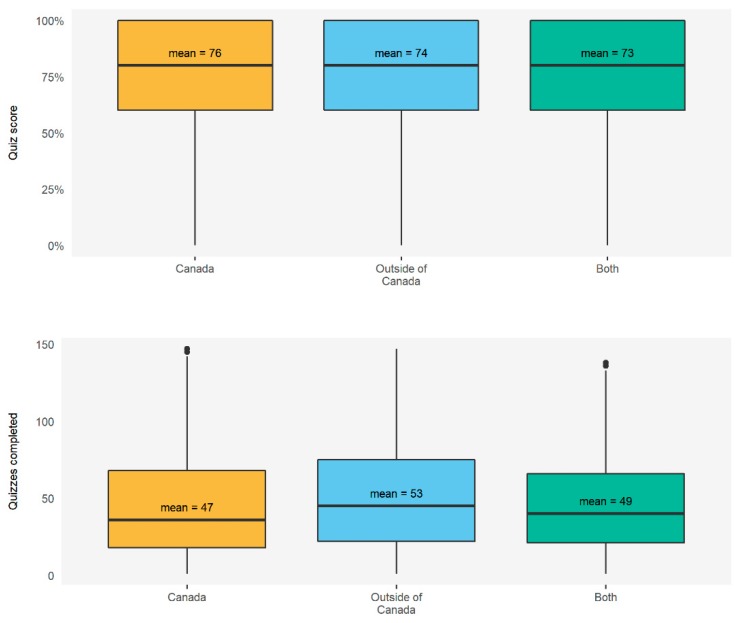
Performance and engagement by location of training.

Users who obtained their entry to practice training in Canada performed better than other users, while engagement was similar whether users trained inside or outside Canada. According to the ANOVA, there were statistically significant differences between training locations for performance (F (2, 98,228) = 98.21, *p* < 0.001) and engagement (F (2, 98,228) = 231.7, *p* < 0.001). As with the other comparisons, the differences in performance were small, while the differences in engagement were larger (Appendix A, Figure A8).

#### 3.2.2. Relationship between Topic Type, Performance, and Engagement

**Figure 3:**
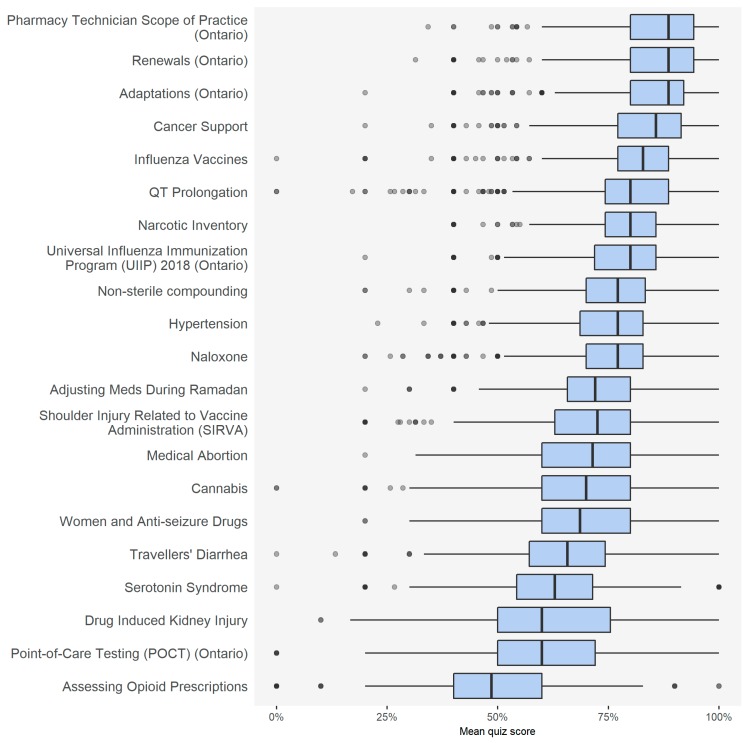
Overall mean quiz score in each module.

The top three modules for user performance were all related to scope of practice (Figure 3). The bottom three modules for performance were all clinical topics. For engagement, the top three modules with the highest completion rate were related to scope of practice, while the modules with the lowest completion rate related to specialty topics (Table 5, Appendix A, Table A1). After adjusting for demographic factors, both user performance and engagement were higher for users who completed more quizzes in 18 of the 21 modules, with the exception of the modules related to Ramadan, cannabis, and non-sterile compounding.

#### 3.2.3. Relationship between Self-Reported Past Behavior and Performance

Overall, users self-reported they had previously completed a behavior 36% of the time, though the number varied according to topic. For example, in the cannabis module, 21% of users responded that they had performed the target behaviors before (e.g., asking a patient about cannabis use, assessing drug therapy for interactions with cannabis medications). By comparison, 44% of users in the renewals module responded they had performed the target behaviors before (e.g., renewing a short- or long-term medication, renewing a medication for longer than a month, renewing a specialist’s prescription). The regression models did not identify any significant differences in performance for users who self-reported that they had previously performed a behavior compared to those who had not, which is also evident from the differences in mean scores (Table 6).

## 4. Discussion

Cluster analysis is a powerful tool for evaluating interventions based on performance and engagement, as it pushes the analysis beyond the limits of traditional statistical methods. In this analysis of a national, computer-based education program, the cluster analysis was useful for identifying clear clusters of users based on their performance and engagement, but it also identified that demographic factors were not overly predictive of who was in each cluster. In this way, the cluster analysis segmented users based on how they interacted with the platform rather than who they were. In contrast, traditional statistics identified several differences in the performance and engagement of different demographic groups, however these differences were generally small. For example, pharmacists who had higher overall quiz scores and who completed more modules were slightly more likely to work in community pharmacies compared to other settings. This likely reflects that the content was developed primarily for community pharmacists. Similarly, pharmacists and technicians who had lower scores or who completed fewer modules were more likely to have trained outside Canada, though these differences were so small that they are unlikely to have a significant impact on program evaluation. This latter finding is important because thirty percent of Canadian pharmacists are trained outside Canada [25]. Thus, there is great interest in identifying if place of training impacts performance and engagement. The results showed that while those who were trained internationally had slightly lower overall scores, they had a similar engagement to those trained in Canada. However, the differences were small and there is a risk that the findings could be misinterpreted to devalue the knowledge and experience of internationally trained pharmacists. Thus, while all of the traditional comparisons are interesting and not unexpected, their small size differences limit their usefulness. In contrast, the cluster analysis clearly identified the clusters of users who struggled with either performance or engagement.

The contrast between performance and engagement could potentially be explained by looking closer at motivation. For example, social motivation theory suggests behavior change depends on both intrinsic and extrinsic motivation [26,27]. For computer-based education, users can be engaged to participate through extrinsic motivators such as rewards and points systems, but eventually users must develop intrinsic motivations to continue to use the system or to change the target behavior. In this study, the most engaged users were pharmacy students, suggesting that they may already have more extrinsic motivations through assessments in the classroom or experiential education sites. Thus, pharmacists in practice may benefit from additional features that extrinsically motivate them to engage with the platform to bring their engagement more in line with pharmacy students. Further, users working in independent pharmacies were more engaged than users in other practice settings such as chain stores, suggesting that pharmacists outside chains may have less access to continuing education (e.g., annual conferences). Thus, independent pharmacists may have more intrinsic motivation to complete the modules whereas chain store employees may need more extrinsic motivators to complete a full module.

One notable finding was that a minority of users had typically offered a service or performed an activity prior to completing the module, and that past experience providing a service did not necessarily predict a higher quiz score. Numerous studies have shown that pharmacists can be trained to effectively adopt new scopes of practice [28]. Yet, as Rosenthal, Austin, and Tsuyuki highlighted in 2010, pharmacists are held back by a fear of responsibility, paralysis when facing ambiguity, risk aversion, and the need for approval [29]. Thus, it is unsurprising that the majority of users who engaged with a module had little to no prior experience with the topic in daily practice. Further, demographic factors were not necessarily predictive of whether someone had engaged in an activity in the past.

While these results are based on a large, national database, there are some limitations. The dataset is limited to quiz and self-report data from single time points. Additional information would be needed to assess impact on sustained knowledge or behavior change. Further, there is likely a self-selection bias where users who are more interested in or comfortable with online education signed up to use the platform. That said, all users who had completed at least one quiz were included to ensure a broad sample of pharmacy professionals for the analysis. Other possible factors that are not captured in the database but that could potentially impact performance and engagement include past experience with online games, educational platforms, and other online experience. More research needs to be done to determine the impacts of the platform on knowledge and behavior change and to explore the impact of technology experience on engagement in computer-based education. 

## 5. Conclusions

Cluster analysis was a useful strategy for analyzing engagement and performance independent of demographics. While it is important to consider demographics like gender and country of training, these variables also lend themselves to bias and cannot be changed. In this study, pharmacists had higher quiz scores than technicians and students, but students were more engaged with the platform. Community pharmacists performed slightly better than hospital pharmacists, but were much more engaged, while internationally trained pharmacists and technicians performed slightly less well, but were as engaged with the platform as others. That said, the cluster analysis using performance and engagement allowed movement beyond demographics to look at user performance regardless of who the users were. It is a promising strategy for evaluating computer-based education interventions on learning.

## Figures and Tables

**Table 1:** User demographics (N = 5290).

	Number (%)	Performance (Mean Score)	Engagement (Mean Quizzes Completed)
Gender Identity			
Female	3739 (71%)	75	18
Male	1538 (29%)	74	19
Non-binary	13 (0.3%)	75	25
Age			
<25 years	551 (10%)	74	16
25–44 years	3082 (58%)	75	17
45–64 years	1538 (29%)	74	22
>64 years	119 (2%)	71	31
Years in Practice			
<10 years	3029 (57%)	75	18
10–19 years	971 (18%)	75	16
>20 years	1290 (24%)	74	23
User Type			
Pharmacist	3579 (68%)	75	20
Technician	595 (11%)	75	13
Pharmacy Student	711 (13%)	75	20
Technician Student	405 (8%)	74	16
Practice Type			
Community (independent)	1326 (25%)	73	20
Community (large chain)	1883 (36%)	75	21
Community (small chain)	768 (15%)	75	20
Primary Care	92 (2%)	76	15
Long Term Care	130 (2%)	76	18
Hospital	935 (18%)	75	12
University/Academia	156 (3%)	74	12
Entry-to-Practice Training			
Canada	4025 (76%)	76	18
International	941 (18%)	72	20
Both	324 (6%)	70	21
Daily Prescription Count			
Median	120		
Mean	156		

**Table 2:** Demographics for the four pharmacist clusters.

	Cluster 1 (N = 1141)	Cluster 2 (N = 727)	Cluster 3 (N = 1142)	Cluster 4 (N = 569)	Overall (N = 3579)
Overall quiz score					
Mean (SD)	81 (8)	57 (11)	79 (9)	76 (6)	75 (13)
Median	80	60	80	76	76
Range	68–100	0–71	57–100	49–90	0–100
Number quizzes completed on the platform					
Mean (SD)	13 (10)	7 (7)	11 (11)	65 (28)	20 (24)
Median	10	4	7	56	9
Range	1–39	1–70	1–51	30–147	1–147
Number of modules attempted					
Mean (SD)	3 (2)	2 (2)	2 (2)	10 (4)	4 (4)
Median	2	1	2	9	2
Range	1–10	1–13	1–11	5–21	1–21
Proportion of quizzes completed per module attempted					
Mean (SD)	87 (15)	72 (27)	31 (15)	71 (27)	64 (31)
Median	94	77	29	79	67
Range	29–100	14–100	14–69	14–100	14–100
Persistence Score (combination of proportion of quizzes and modules completed)					
Mean (SD)	50 (7)	41 (13)	21 (8)	60 (14)	41 (18)
Median	52	44	20	62	43
Range	35–71	10–81	10–37	26–95	10–95
Gender					
Male	333 (29%)	263 (36%)	397 (35%)	166 (29%)	1159 (32%)
Female	808 (71%)	463 (64%)	741 (65%)	400 (70%)	2412 (67%)
Other	0 (0%)	1 (0%)	4 (0%)	3 (1%)	8 (0%)
Year of birth					
Mean (SD)	1978 (11)	1976 (12)	1978 (11)	1973 (13)	1977 (12)
Median	1980	1978	1980	1972	1979
Range	1918–2001	1918–2001	1918–2001	1918–2001	1918–2001
Highest level of education					
Bachelor in Pharmacy	837 (74%)	532 (74%)	848 (75%)	448 (79%)	2665 (75%)
Entry Level PharmD	143 (13%)	71 (10%)	140 (12%)	53 (9%)	407 (11%)
Graduate PharmD	63 (6%)	39 (5%)	59 (5%)	23 (4%)	184 (5%)
Masters	81 (7%)	61 (8%)	78 (7%)	40 (7%)	260 (7%)
PhD	13 (1%)	18 (2%)	12 (1%)	5 (1%)	48 (1%)
Did not answer	4	6	5	0	15
Location of entry-to-practice training					
Canada	823 (72%)	422 (58%)	813 (71%)	376 (66%)	2434 (68%)
International/Both	318 (28%)	305 (42%)	329 (29%)	193 (34%)	1145 (32%)
Year started practicing					
Mean (SD)	2005 (11)	2004 (12)	2005 (12)	2001 (14)	2004 (12)
Median	2009	2008	2009	2004	2008
Range	1969–2019	1971–2019	1969–2019	1969–2019	1969–2019
Type of pharmacy practice					
Hospital	181 (16%)	134 (18%)	201 (18%)	48 (8%)	564 (16%)
Community	888 (78%)	557 (77%)	880 (77%)	503 (88%)	2828 (79%)
Primary care	36 (3%)	12 (2%)	22 (2%)	8 (1%)	78 (2%)
Long term care	31 (3%)	16 (2%)	33 (3%)	10 (2%)	90 (3%)
University	5 (0%)	8 (1%)	6 (1%)	0 (0%)	19 (1%)
Average number of prescriptions per shift					
Mean (SD)	156 (150)	158 (234)	152 (136)	143 (107)	153 (161)
Median	120	120	120	120	120
Range	0–1700	0–3500	0–1500	0–1000	0–3500
Not applicable	721	438	727	315	2201

**Table 3:** Demographics for the four technician clusters.

	Cluster 1 (N = 97)	Cluster 2 (N = 94)	Cluster 3 (N = 196)	Cluster 4 (N = 208)	Overall (N = 595)
Overall quiz scores					
Mean (SD)	51 (11)	71 (8)	80 (10)	83 (8)	75 (15)
Median	51	72	80	82	77
Range	20–66	48–88	60–100	64–100	20–100
Overall quizzes completed					
Mean (SD)	4 (4)	43 (22)	8 (7)	8 (6)	13 (16)
Median	2	35	7	7	7
Range	1–17	16–123	1–33	1–28	1–123
Persistence					
Mean (SD)	40 (15)	51 (15)	19 (8)	48 (8)	37 (17)
Median	44	50	17	52	38
Range	10–57	21–90	10–35	31–64	10–90
Proportion of quizzes completed					
Mean (SD)	72 (29)	67 (27)	29 (14)	87 (16)	62 (32)
Median	79	64	29	98	64
Range	14–100	14–100	14–64	43–100	14–100
Number of modules attempted					
Mean (SD)	1 (1)	7 (3)	2 (1)	2 (1)	3 (3)
Median	1	6	1	1	2
Range	1–4	3–17	1–7	1–7	1–17
Gender					
Male	11 (11%)	10 (11%)	20 (10%)	11 (5%)	52 (9%)
Female	86 (89%)	84 (89%)	176 (90%)	196 (94%)	542 (91%)
Other	0 (0%)	0 (0%)	0 (0%)	1 (0%)	1 (0%)
Year of birth					
Mean (SD)	1980 (10)	1978 (12)	1978 (12)	1979 (10)	1978 (11)
Median	1980	1977	1979	1980	1979
Range	1958–1997	1956–1999	1918–2001	1953–1997	1918–2001
Location of entry-to-practice training					
Canada	91 (94%)	93 (99%)	192 (98%)	204 (98%)	580 (97%)
International/Both	6 (6%)	1 (1%)	4 (2%)	4 (2%)	15 (3%)
Year started practicing					
Mean (SD)	2007 (9)	2006 (12)	2005 (11)	2007 (10)	2006 (10)
Median	2009	2012	2008	2010	2010
Range	1977–2019	1979–2019	1976–2019	1979–2018	1976–2019
Type of pharmacy					
Hospital	52 (54%)	30 (32%)	95 (48%)	106 (51%)	283 (48%)
Community	42 (43%)	55 (59%)	90 (46%)	96 (46%)	283 (48%)
Primary care	0 (0%)	0 (0%)	0 (0%)	0 (0%)	0 (0%)
Long term care	3 (3%)	9 (10%)	9 (5%)	6 (3%)	27 (5%)
University	0 (0%)	0 (0%)	2 (1%)	0 (0%)	2 (0%)
Average number of prescriptions per shift					
Mean (SD)	330 (617)	187 (139)	234 (433)	260 (305)	252 (394)
Median	200	175	150	200	200
Range	0–3000	0–500	0–3000	0–2200	0–3000
Not applicable	67	64	149	144	424

**Table 4:** Relationship between performance (quiz score), engagement (quizzes completed), and user demographics (98,231 observations).

	Performance (Overall Quiz Score)	Engagement (Number of Quizzes Completed)
Variable	Estimate (Std. error)	Estimate (Std. error)
User type (reference pharmacist)		
Pharmacy student	−0.039 * (0.011)	0.262* (0.008)
Pharmacy technician	−0.152 * (0.013)	−0.268 * (0.010)
Pharmacy technician student	−0.363 * (0.013)	−0.032 * (0.010)
Gender (reference male)		
Female	0.046 * (0.007)	0.026 (0.006)
Other	0.028 (0.059)	0.297 * (0.044)
Location of training (reference Canada)		
Outside Canada	−0.126 * (0.009)	0.007 (0.007)
Both	−0.231 * (0.013)	−0.108 * (0.010)
Practice type (reference hospital)		
Independent	0.014 (0.012)	0.331 * (0.009)
Large chain	0.070 * (0.011)	0.257 * (0.009)
Small chain	0.058 * (0.013)	0.232 * (0.010)
Primary care	0.166 * (0.031)	0.178 * (0.022)
Long term care	0.074 * (0.024)	0.237 * (0.018)
University	0.101 * (0.027)	−0.136 * (0.020)
Year started practicing Year of birth	0.002 * (0.000) 0.001 * (0.000)	−0.001 * (0.000) −0.032 * (0.000)

* denotes statistically significant estimates.

**Table 5:** Relationship between quiz score (performance) and quizzes completed (engagement) across all modules.

Module	Mean Quiz Score	Mean Module Completion Rate	Quizzes Completed Estimate (std. Error)	Quiz Score Estimate (std. Error)
Adaptations (Ontario)	87%	67%	0.126 * (0.01)	0.194 * (0.013)
Adjusting Meds During Ramadan	72%	52%	−0.005 (0.012)	−0.01 (0.017)
Assessing Opioid Prescriptions	52%	43%	0.083 * (0.008)	0.237 * (0.014)
Cancer Support	85%	57%	0.138 * (0.013)	0.307 * (0.02)
Cannabis	68%	47%	−0.068 * (0.006)	−0.119 * (0.008)
Drug-Induced Kidney Injury	64%	53%	0.126 * (0.011)	0.211 * (0.014)
Hypertension	76%	56%	0.022 * (0.01)	0.042 * (0.014)
Influenza Vaccines	83%	71%	0.083 * (0.01)	0.097 * (0.012)
Medical Abortion	72%	66%	0.052 * (0.015)	0.07 * (0.018)
Naloxone	76%	65%	0.029 * (0.009)	0.045 * (0.011)
Narcotic Inventory	78%	53%	0.012 (0.011)	0.026 (0.017)
Non-sterile compounding	76%	52%	−0.018 * (0.009)	−0.036 * (0.013)
Pharmacy Technician Scope of Practice (Ontario)	87%	69%	0.052 * (0.012)	0.081 * (0.016)
Point-of-Care Testing (Ontario)	64%	45%	0.089 * (0.007)	0.262 * (0.013)
QT Prolongation	81%	54%	0.055 * (0.009)	0.129 * (0.014)
Renewals (Ontario)	87%	68%	0.149 * (0.01)	0.214 * (0.014)
Serotonin Syndrome	64%	50%	0.063 * (0.007)	0.134 * (0.01)
Shoulder Injury Related to Vaccine Administration (SIRVA)	72%	63%	0.078 * (0.008)	0.124 * (0.01)
Travellers’ Diarrhea	65%	57%	0.034 * (0.01)	0.065 * (0.014)
Universal Influenza Immunization Program (UIIP) 2018 (Ontario)	80%	71%	0.086 * (0.01)	0.092 * (0.011)
Women and Anti-seizure Drugs	70%	49%	0.051 * (0.018)	0.121 * (0.028)

* denotes statistically significant estimates.

**Table 6:** Proportion of users who indicated that they had performed the target behaviors across modules.

Module	Users Reporting They Performed the Target Behavior	Mean Score for Users Who Did Not Perform the Behavior	Mean Score for Users Who Did Perform the Behavior
Adaptations (Ontario)	43%	90%	88%
Adjusting Meds During Ramadan	20%	70%	65%
Assessing Opioid Prescriptions	24%	55%	58%
Cancer Support	31%	87%	88%
Cannabis	21%	65%	64%
Drug-induced Kidney Injury	28%	62%	68%
Hypertension	34%	75%	79%
Influenza Vaccines	38%	83%	88%
Medical Abortion	20%	74%	73%
Naloxone	30%	76%	72%
Narcotic Inventory	40%	75%	82%
Non-sterile compounding	41%	75%	75%
Pharmacy Technician Scope of Practice (Ontario)	35%	86%	88%
Point-of-Care Testing (POCT) (Ontario)	14%	67%	65%
QT Prolongation	38%	81%	81%
Renewals (Ontario)	44%	88%	88%
Serotonin Syndrome	35%	61%	68%
Shoulder Injury Related to Vaccine Administration (SIRVA)	42%	74%	68%
Travellers’ Diarrhea	27%	65%	62%
Universal Influenza Immunization Program (UIIP) 2018 (Ontario)	38%	76%	85%
Women and Anti-seizure Drugs	25%	67%	71%

**Figure A9:**
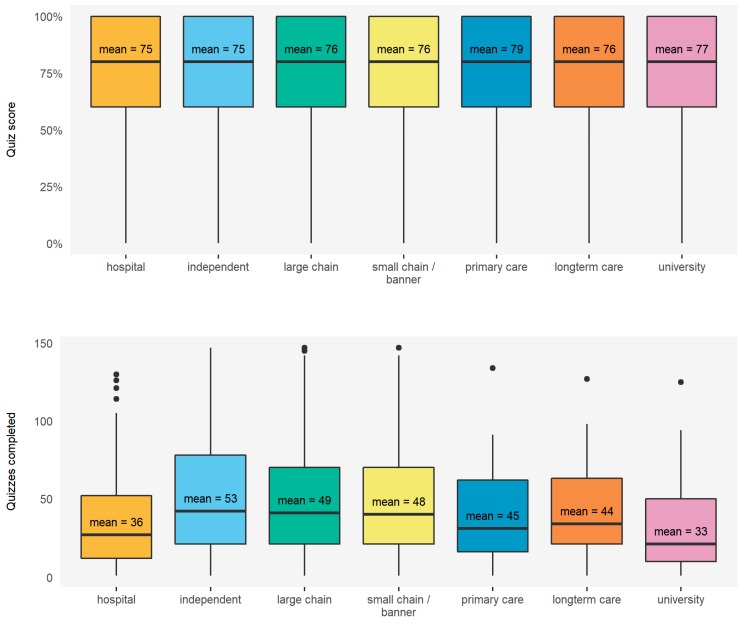
Performance and engagement by practice type.

## Appendix A

The following histograms show the distribution of users based on the number of quizzes completed, quizzes attempted, and the overall quiz mean score.

Relationships between performance, engagement and user demographics

To understand the relationship between different demographic factors and users’ performance, the following binomial regression model was built:

*Correct responses per quiz = intercept + a1 * user type + a2 * gender + a3 * location of training + a4 * practice type + a5* year started practicing + a6 * year of birth.*

To understand the relationship between different demographic factors and users’ engagement, the following negative binomial regression model was built:

*Quizzes completed = intercept + a1 * user type + a2 * gender + a3 * location of training + a4 * practice type + a5* year started practicing + a6 * year of birth.*

The range of the number of quizzes completed (1 to 147) is large and these data are over-dispersed (variance is much larger than the mean). Therefore, a traditional Poisson model for counts of quizzes would not be appropriate here.

The results of these models are given in Table 4.

Based on the regression models, pharmacists performed better than all other user categories and pharmacy students had the highest level of engagement.

Investigation of the differences in performance and engagement for certain demographic factors individually was wanted. Univariate ANOVA models were used, followed by Tukey’s honest significance differences test to look for statistically significant differences between groups.

Based on the regression models, pharmacists performed better than all other user categories and pharmacy students had the highest level of engagement (Table 4). According to the ANOVA, there were statistically significant differences between user categories for performance (F (3, 98,227) = 117.8, *p* < 0.001) and engagement (F(3, 98,227) = 623.4, *p* < 0.001). The differences in performance were small, while the differences in engagement were more pronounced (Appendix A, Figure A7).

Users who obtained their entry to practice training in Canada performed better than other users, while engagement was similar whether users trained inside or outside Canada (Table 4). According to the ANOVA, there were statistically significant differences between training locations for performance (F (2, 98228) = 98.21, *p* < 0.001) and engagement (F (2, 98,228) = 231.7, *p* < 0.001). However, as above, the differences in performance were small, while the differences in engagement were more pronounced (Appendix A, Figure A8).

Users working in primary care performed better than other users, while engagement was highest for users working in independent practice (Table 4). According to the ANOVA, there were statistically significant differences between practice locations for performance (F (6, 98,224) = 17.58, *p* < 0.001) and engagement (F (6, 98,224) = 372.2, *p* < 0.001). The differences in performance were small, while the differences in engagement were more pronounced (Appendix A, Figure A9).

**Table A1:** Module completion rate.

	Users Attempting Module	Users Completing Module	Module Completion Rate
Adaptations (Ontario)	1318	877	67%
Adjusting Meds During Ramadan	523	270	52%
Assessing Opioid Prescriptions	807	343	43%
Cancer Support	569	323	57%
Cannabis	1574	738	47%
Drug-Induced Kidney Injury	532	281	53%
Hypertension	748	416	56%
Influenza Vaccines	1166	825	71%
Medical Abortion	392	257	66%
Naloxone	1087	710	65%
Narcotic Inventory	634	336	53%
Non-sterile compounding	1019	532	52%
Pharmacy Technician Scope of Practice (Ontario)	958	663	69%
Point-of-Care Testing (POCT) (Ontario)	986	441	45%
QT Prolongation	1104	593	54%
Renewals (Ontario)	1118	760	68%
Serotonin Syndrome	1134	565	50%
Shoulder Injury Related to Vaccine Administration (SIRVA)	1309	823	63%
Travellers’ Diarrhea	676	388	57%
Universal Influenza Immunization Program (UIIP) 2018 (Ontario)	1028	730	71%
Women and Anti-seizure Drugs	202	99	49%

One way to determine if there was a relationship between user performance and engagement was to model quiz scores as a function of quizzes completed. Twenty-one binomial regression models (one for each module) were built with the following structure:

*Correct responses per quiz = intercept + a* quizzes completed + b1 * user type + b2 * gender + b3 * location of training + b4 * practice type + b5* year started practicing + b6 * year of birth.*

Another way to determine the relationship between user performance and engagement, was to model quizzes completed as a function of quiz scores. Twenty-one Poisson regression models (one for each module) were built with the following structure:

*Quizzes completed = intercept + a* correct responses per quiz + b1 * user type + b2 * gender + b3 * location of training + b4 * practice type + b5* year started practicing + b6 * year of birth.*

The results of these models are given in Table 5.
